# QTL meta-analysis of root traits in *Brassica napus* under contrasting phosphorus supply in two growth systems

**DOI:** 10.1038/srep33113

**Published:** 2016-09-14

**Authors:** Ying Zhang, Catherine L. Thomas, Jinxia Xiang, Yan Long, Xiaohua Wang, Jun Zou, Ziliang Luo, Guangda Ding, Hongmei Cai, Neil S. Graham, John P. Hammond, Graham J. King, Philip J. White, Fangsen Xu, Martin R. Broadley, Lei Shi, Jinling Meng

**Affiliations:** 1 National Key Laboratory of Crop Genetic Improvement and National Centre of Plant Gene Research, Huazhong Agricultural University, Wuhan 430070, China; 2 Key Laboratory of Arable Land Conservation (Middle and Lower Reaches of Yangtze River), Ministry of Agriculture, Huazhong Agricultural University, Wuhan 430070, China; 3 Plant and Crop Sciences Division, School of Biosciences, University of Nottingham, Sutton Bonington Campus, Loughborough LE12 5RD, United Kingdom; 4 Biotechnology Research Institute, Chinese Academy of Agricultural Sciences, Beijing 100081, China; 5 School of Agriculture, Policy and Development, University of Reading, Reading RG6 6AR, United Kingdom; 6 Southern Cross Plant Science, Southern Cross University, Lismore NSW 2480, Australia; 7 The James Hutton Institute, Invergowrie, Dundee DD2 5DA, United Kingdom; 8 King Saud University, Riyadh 11451, Kingdom of Saudi Arabia

## Abstract

A high-density SNP-based genetic linkage map was constructed and integrated with a previous map in the Tapidor x Ningyou7 (TNDH) *Brassica napus* population, giving a new map with a total of 2041 molecular markers and an average marker density which increased from 0.39 to 0.97 (0.82 SNP bin) per cM. Root and shoot traits were screened under low and ‘normal’ phosphate (Pi) supply using a ‘pouch and wick’ system, and had been screened previously in an agar based system. The P-efficient parent Ningyou7 had a shorter primary root length (PRL), greater lateral root density (LRD) and a greater shoot biomass than the P-inefficient parent Tapidor under both treatments and growth systems. Quantitative trait loci (QTL) analysis identified a total of 131 QTL, and QTL meta-analysis found four integrated QTL across the growth systems. Integration reduced the confidence interval by ~41%. QTL for root and shoot biomass were co-located on chromosome A3 and for lateral root emergence were co-located on chromosomes A4/C4 and C8/C9. There was a major QTL for LRD on chromosome C9 explaining ~18% of the phenotypic variation. QTL underlying an increased LRD may be a useful breeding target for P uptake efficiency in *Brassica*.

Phosphorus (P) is an essential macronutrient for plant growth involved in many key metabolic pathways. However, 80% of P content in soil can be fixed in forms unavailable to plants^1^. Hence, P is one of the major limitations for crop productivity worldwide. Enhancing P uptake and utilization efficiency (plant biomass per unit input of P) through breeding and genetic technology is an important strategy to reduce the application of phosphate (Pi) fertilizers to crops. Resolving P efficiency to sub-traits important for P uptake provides a more targeted strategy than selecting for varieties with improved yield under P deficiency, because field settings are complex and the results from one may not be reproducible in another^2^,^3^,^4^,^5^.

Phosphorus deficiency in plants leads to changes in root morphology and architecture, including the formation of longer, thinner roots, increased lateral root production and growth, cluster root formation, and greater root hair length and density^3^,^6^,^7^,^8^,^9^. Phosphorus is an immobile resource which remains largely in the top soil^1^. Consequently, shallow root angles conferring better top-soil foraging have been shown to increase growth under P deficiency^3^,^8^,^10^. To adapt to P stress, it is observed that plants alter their allocation of biomass to roots rather than shoots, thus the root/shoot ratio increases^11^,^12^.

P efficiency is controlled by complex quantitative traits. Many root traits and QTL associated with P uptake efficiency have been identified. For example, QTL have been found for root hair growth in common bean (*Phaseolus vulgaris*)^13^ and maize^14^, decreased basal root gravitropism in common bean^15^ and adventitious rooting which has greater horizontal growth and specific root length compared to basal rooting^16^. *Pup1*/*PSTOL1* was the first P efficiency QTL/gene to be introgressed into a P intolerant variety of rice (*Oryza sativa*) and enhance grain yield in P deficient soils, which functioned to keep normal development of the root under P deficiency^17^,^18^. Greater basal root whorl number affords a greater vertical range of soil exploration of common bean^10^. In *B. oleracea*, accessions with greater yield under low P conditions had greater lateral root number, length and growth rate^19^. In *B. napus*, the P-efficient genotype Eyou Changjia developed a larger root system than the P-inefficient genotype B104-2^20^. A *B. napus* RIL population derived from a cross between Eyou Changjia and B104-2 showed that low P specific yield-related QTLs were clustered on chromosomes A1, A6 and A8^21^. In the *B. napus* TNDH population, low-P specific yield-related QTL were observed in field trials clustered on A2, A3 and A5^22^. Yet, to date no root trait QTL have been introgressed into commercial varieties of *Brassica,* in part due to limited validation of conserved QTL across populations and environments^23^,^24^, and poorly resolved chromosomal regions^4^.

High throughput phenotyping (HTP) has become a priority for crop breeding research in order to obtain relevant root trait data. However, since large scale field screening remains costly, time demanding and inefficient, obtaining accurate phenotype data is a bottleneck for crop root research. Therefore, various 2D root HTP systems have been deployed, including agar plate-based systems^25^,^26^,^27^ and ‘pouch and wick’ systems, in which roots are grown on filter paper, suspended in solution, which is surrounded by a pouch which conceals roots from light^5^,^10^,^28^,^29^,^30^,^31^.

High-density genetic linkage maps are prerequisites for map-based cloning and marker-assisted plant breeding. In recent years, with the development of the next-generation sequencing and SNP genotyping arrays, SNPs have become the marker of choice in most species for construction of high density genetic linkage maps such as rice and maize^32^,^33^. SNP discovery is challenging in allopolyploid species such as *B. napus*, as they may arise both between allelic (homologous) sequences within subgenomes and between homoeologous sequences among subgenomes, as well as from polymorphisms between paralogous duplicated sequences^34^. In 2011 an international *Brassica* SNP consortium produced an Infinium^TM^ genotyping array, with over 50,000 (60 k) SNPs, for *B. napus*, in cooperation with Illumina Inc. (San Diego, CA, USA)^35^. This introduced a very efficient method for high-density, sequence-based, genome-wide polymorphism screening of *B. napus* populations. For example, Liu *et al.* (2013) constructed the first SNP genetic map with the 60 k SNP chip of *B. napus*, which contains 9164 SNP markers covering 1832.9 cM with an average distance of 0.66 cM between adjacent markers^36^.

Oilseed rape (OSR, Canola, *Brassica napus* L.) is a globally important source of oil and provides 18% of global vegetable oil for human consumption, industrial oils, biodiesel, lubricant, and fodder for animal feeds. Over 70 million tonnes of rapeseed are produced annually worldwide^37^ (FAOSTAT). In this study, a high-density TNDH SNP-based genetic linkage map of *B. napus* was constructed. QTLs controlling root architecture and shoot traits under low Pi (LP) and ‘normal’ Pi (NP) conditions were identified using a hydroponic ‘pouch and wick’ system^5^,^31^, and were integrated with those from an agar based screening system^27^ in a meta-analysis. This analysis will investigate conserved QTL for root traits, and reduce the corresponding confidence interval, thus refining the chromosomal region of useful root loci.

## Results

### Construction of a high-density SNP based genetic linkage map

A total of 30,717 SNP markers scored from the Brassica 60 k array showed polymorphisms between Tapidor and Ningyou7. Of these, the 13,753 SNP markers with ≤5% missing data were used for clustering the ‘genetic bins’, conducting genetic linkage analysis and linkage map construction. SNP bin markers specific to the *B. napus* A and C genomes were clearly separated into different linkage groups at LOD 4–19, respectively. A set of 1,826 SNP bins, consisting of 13,612 SNP markers, were successfully assigned to 19 linkage groups representing the A1-A10 and C1-C9 linkage groups of *B. napus*, with total genetic distances of 913.7 cM and 1004.2 cM for the A and C genomes, respectively. The marker number and density varied considerably across different chromosomes. The map was named the ‘1826-map’ (Table S1).

**Figure 1:**
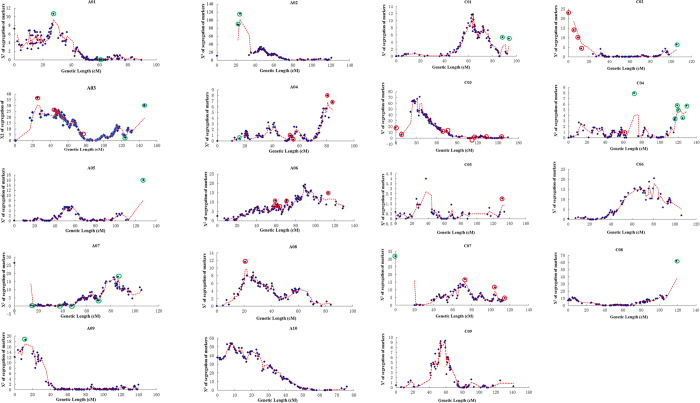
The segregation of markers on each linkage group of the *Brassica napus* TNDH 2109-map. Chi-squared test: n = 2, df (degrees of freedom) = 1, P = 0.05, χ^2^ = 3.84, SD* = segregation distortion. When the segregation of markers was 3.84 the segregation ratio of Tapidor and Ningyou7 was 1:1. Red and green circles indicate the extreme markers skewed towards Tapidor and Ningyou7, respectively, which were deleted to construct the new 2041 map.

An integrated genetic linkage map named the ‘2109-map’ was constructed (Table S1), which had 19 linkage groups and a length of 2210.2 cM, with a total of 2109 molecular markers, comprising 1734 SNP bins from the 1826-map and 375 original markers (SSR, RFLP, SNP and STS) derived from the previous TNDH genetic linkage map with 798 markers (798-map)^22^,^27^. Among the 2109 markers, 916 (43.4%) showed genetic distortion (P < 0.05). Thus, of the total segregation distortion (SD) markers, 433 and 483 markers were skewed towards the female parent Tapidor and male parent Ningyou7, respectively. The SD markers were detected on all chromosomes (Fig. 1). The extreme SD markers which significantly deviated from the fit-line in the 2109-map were removed. At the same time, some markers associated with these extreme SD markers were also absent in the newest 2041-map (Fig. 1; Table 1). 60.1% and 39.9% of the 2041 markers were mapped on the A genome and C genome, respectively. The marker density was 1.18 per cM on the A genome, 0.76 per cM on C genome and 0.97 per cM over the whole genome. The number and density of SNP bins on the A genome were significantly greater than those on the C genome in all four maps (Table S1; Table 1). Average density increased from 0.39 markers per cM on the 798-map to 0.95 markers (0.95 SNP bin) per cM on the 1826-map and 0.97 marker (0.82 SNP bin) per cM on 2041-map (Table S1; Table 1). Moreover, perfect collinearity was observed in the alignment of these markers on the three genetic linkage maps, except for a small number of inversions.

### Root and shoot traits of seedlings grown in the ‘pouch and wick’ system

**Figure 2:**
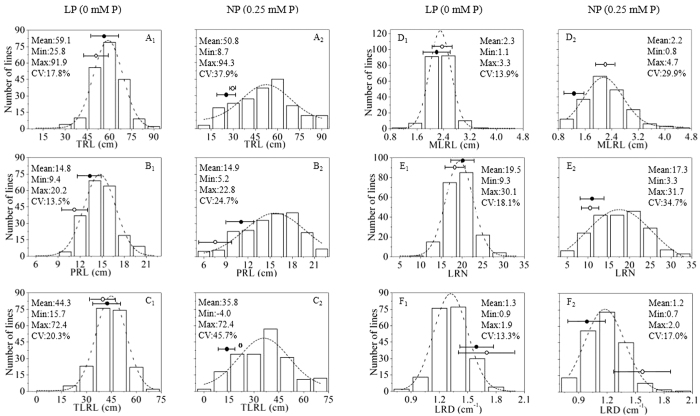
Frequency distribution of root architectural traits of the TNDH population grown for 14 days in a ‘pouch and wick’ system under low Pi (LP, 0 mM P) and ‘normal’ Pi (NP, 0.25 mM P). A = TRL (total root length); B = PRL (primary root length); C = TLRL (total lateral root length); D = MLRL (mean lateral toot length); E = LRN (lateral root number); F = LRD (lateral root density). Open circles = parent Ningyou7; filled circles = parent Tapidor.

All root traits showed approximately normal distribution and transgressive segregation at both LP and NP, with means under LP ranging from: PRL 9.4–20.2 cm; TRL 25.8–91.9 cm; TLRL 15.7–72.4 cm; MLRL 1.1–3.3 cm; LRN 9.3–31.0; LRD 0.9–1.9 cm^−1^, at LP (Data S1; Fig. 2).

All root traits, except PRL, showed significantly greater growth under the LP as compared with the NP condition (*p* < 0.001) (Fig. 2; Data S2). However, REML variance components analysis showed a greater effect of genotype than treatment, with the genotype effect (a measure of broad-sense heritability) ranging from 2% in MLRL to 11% in PRL, the treatment effect ranging from 0% in MLRL to 5% in LRD (Data S3). Thus, relationships across the LP and NP treatments showed significant positive correlations in PRL, LRN and LRD (*p* < 0.05) (Data S2). Within both LP and NP treatments, all root traits were significantly positively correlated (*p* < 0.001- *p* < 0.05), except for significant negative correlations between PRL and LRD (*p* < 0.001), MLRL and LRD (*p* < 0.001) (LP only), MLRL and LRN (*p* < 0.001) and PRL and MLRL (*p* < 0.001) (NP only) (Data S2).

**Figure 3:**
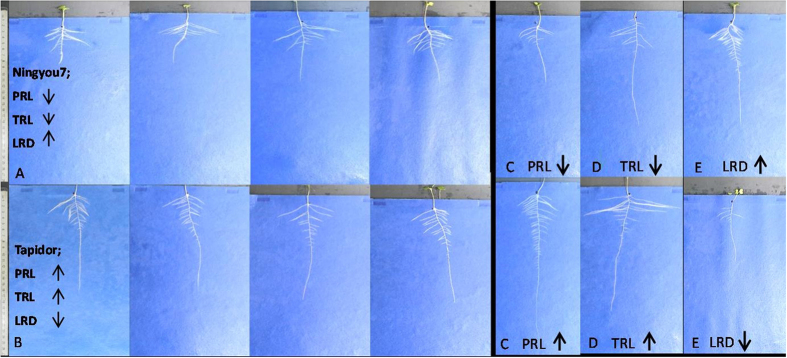
Illustrative examples of parents (**A**) Ningyou7 (**B**) Tapidor and the extreme genotypes with the mean minimum and maximum (**C**) PRL (TN52, TN15, respectively), (**D**) TRL (TN58 and TN38, respectively) and (**E**) LRD (TN40 and TN53), at 14 DAS having grown in a ‘pouch and wick’ system under a low Pi treatment (LP, 0 mM Pi). Primary root length (PRL), total root length (TRL), lateral root density (LRD), scale = 1 cm.

The parent Tapidor as compared with Ningyou7 had significantly greater TRL, PRL, TLRL and LRN (*p* < 0.001), but a shorter MLRL and a significantly lower LRD (*p* < 0.05) under LP. Likewise, under NP Tapidor had a longer PRL and a significantly lower LRD compared to Ningyou7 (*p* < 0.05). Under LP, Ningyou7 also had a greater shoot dry weight than Tapidor (*p* < 0.01) and a greater shoot/root ratio (*p* < 0.01) (Data S2; Figs 2 and 3).

### QTL and meta-analysis of root and shoot traits grown under LP and NP in the ‘pouch and wick’ system

**Figure 4:**
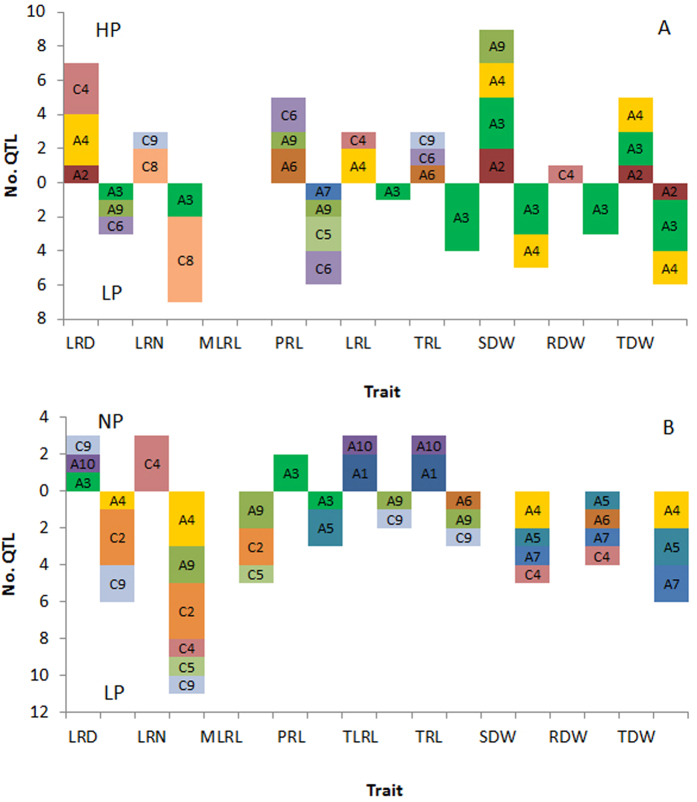
QTL above the LOD threshold of 2.5 for root/shoot traits in (**A**) seedlings grown for 12 d in the agar system, under HP (0.625 mM Pi, above horizontal) and LP (0 mM Pi, below horizontal) and (**B**) seedlings grown for 14 d in the pouch and wick system, under NP (0.25 mM Pi, above horizontal) and LP (0 mM Pi, below horizontal), on chromosomes A1-10 and C1-9. LRD = lateral root density, LRN = lateral root number, MLRL = mean lateral root length, PRL = primary root length, LRL = total lateral root length, TRL = total root length, SDW = shoot dry weight, RDW = root dry weight, TDW = total dry weight.

Using the 2041 map, a total of 60 QTL were identified from the ‘pouch and wick’ system for root and shoot traits; 46 under LP and 14 under NP, which were located across 13 of the 19 chromosomes (excluding A2, A8, C1, C3, C6 and C7). At LP most QTL were on A9 and C2- 35% and at NP most QTL were on A1- 29%. Considering both Pi treatments together, most QTL for LRD were on C2 and C9- 75%, for LRN most QTL were on A4, C4 and C2- 60%, for MLRL most QTL were on A9 and C2- 67%, for TLRL and TRL most QTL were on A1- 40% and for dry weight most QTL were on A4, A5 and A7- 80%. There were eleven QTL which explained >10% of the phenotypic variance, most of which were on A9/C9 for LRD/LRN, a QTL for LRD on C9 under LP explaining the greatest variance- 17.6%. The positive allelic effect of the majority of QTL came from Tapidor, except all those for PRL and the majority for MLRL from Ningyou7 (Fig. 4; Table S2).

**Figure 5:**
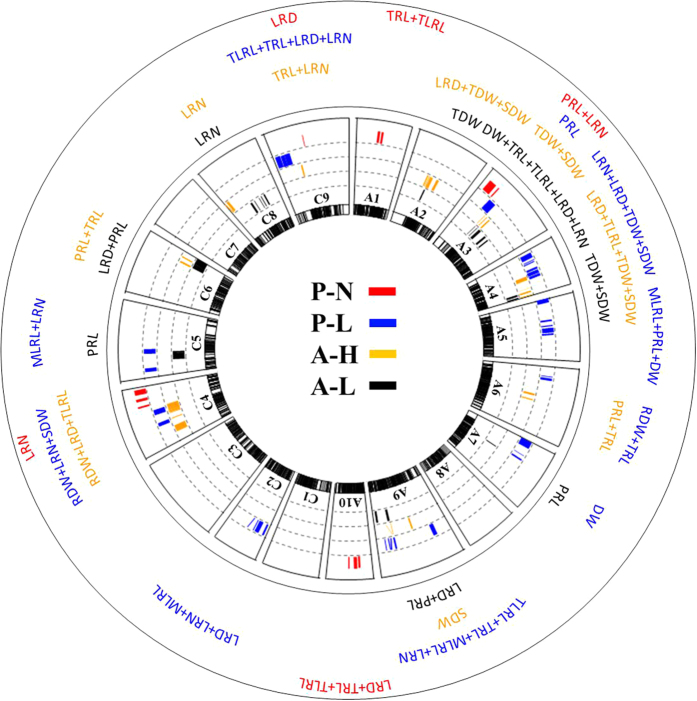
Individual and combined QTL for root and shoot traits within a treatment, shown as colorful bars, with thickness indicating confidence interval, and position indicating location along the chromosome (A1-A10, C1-C9). Red = ‘normal’ Pi (NP, 0.25 mM Pi) and blue = low Pi (LP, 0 mM Pi) in the hydroponic ‘pouch and wick’ system (P), yellow = high Pi (HP, 0.625 mM Pi) and black = low P (LP, 0 mM Pi) in the agar screening system (**A**). LRD = lateral root density, LRN = lateral root number, MLRL = mean lateral root length, PRL = primary root length, TLRL = total lateral root length, TRL = total root length, SDW = shoot dry weight, RDW = root dry weight, TDW = total dry weight. DW means QTL for SDW, RDW and TDW. QTLs were detected using the new TNDH 2041-map.

The QTL within a treatment which had over-lapping confidence intervals (CI) were integrated into combined QTL. Fourteen combined QTL were identified (28 of the original 60 QTL were integrated); at LP there were two combined QTL on A4, one each on A5, A7, and A9, three on C2, one on C4 and two on C9. At NP there was one combined QTL on A3 and two on A1. Most combined QTL were on C2 for LRN/LRD. Confidence intervals were reduced by between 0.9–6.6 cM, a ~33% reduction in combined QTL compared to component QTL (Fig. 5; Table S2).

### QTL and meta-analysis of root and shoot traits grown under LP and HP in the agar system

Using the 2041 map, a total of 71 QTL for root and shoot traits were detected using the data previously collected in an agar screen; 35 under LP and 36 under HP, which were located across 11 of 19 chromosomes (excluding A1, A5, A8, A10, C1, C2, C3 and C7). At LP most QTL were on A3- 49% and under HP most were on A4- 25%. Considering both Pi treatments together, most QTL for LRD were on A4 and C4- 60%, for LRN most QTL were on C8- 70%, for PRL most QTL were on C6- 40%, for TLRL most QTL were on A4- 50%, for TRL most QTL were on A3- 63% and for dry weight most QTL were on A3- 48%. There were 22 major QTLs which explained >10% of the phenotypic variance, most were on A3 and were for shoot dry weight, a QTL on C6 for PRL under HP explaining the greatest variance- 17.5%. The positive allelic effect of the majority of QTLs came from Ningyou7, this was consistent across all traits, except PRL where the most positive effect was from Tapidor (Fig. 4; Table S3).

The QTL within a treatment which had over-lapping CI were integrated into combined QTL. Fourteen combined QTL were identified (35 of the original 71); at LP there were five combined QTL on A3 and two on A4, at HP there were two combined QTL on A3, two on A4, and one each on A6, C6 and C9. Confidence intervals were reduced by between 0.0 – 4.8 cM, a ~56% reduction in combined QTL compared to the component QTL (Fig. 5; Table S3).

### QTL meta-analysis of root and shoot traits across Pi treatments and growth systems

Combined from across the LP and NP treatments under the ‘pouch and wick’ system there was one QTL with over-lapping confidence intervals (although it was not a combined QTL) for PRL on A3. Under the agar system there were seven combined QTL from across the P treatments; TDW under HP and LP on A2, TDW + SDW at HP and RDW + TDW + SDW + TRL at LP on A3, TDW + SDW at HP and LRL + RDW + TDW + SDW + TRL at LP on A3; and RDW + TRL at LP and SDW at HP also on A3, TDW + SDW at LP and LRD + LRL  + TDW + SDW at HP on A4, SDW + TDW at LP and LRL at HP also on A4, LRD at LP and PRL at HP and LP on C6 and PRL at LP and TRL + PRL at HP also on C6. Thus, most combined QTL across treatments were on A3 and integrated QTL for both root traits and dry weight (Fig. 5; Table S4).

Across the pouch and wick and agar based systems there were four combined QTL; for LRN at LP and LRD at HP on A4, MLRL at LP and LRD at LP on A9, SDW at LP and LRD at HP on C4, and LRN at LP and PRL at LP on C5, in the pouch and agar based systems, respectively. The confidence interval reduced from a mean of 7 cM before integration to 4.4 cM after- a reduction of ~35% (Fig. 5; Table S4).

## Discussion

Genetic linkage maps are highly valuable tools for studying genome structure and evolution, comparative genome analyses and localizing genes of interest. In this study, a high density SNP-based genetic linkage map of *B. napus* was constructed (Table 1). The number of markers mapped onto the A genome was greater than that onto the C genome (Table 1; Table S1), as in previous reports^36^,^38^,^39^. This could be attributed to genetic differences between the two parental lines. Asiatic *B. napus* cultivars such as Ningyou7 include more introgressed alleles in the A genome from the genetic background of Chinese *B. rapa*, which could contribute to increased genetic diversity and hence SNP polymorphisms compared to European oilseed varieties^40^,^41^. For instance, the *B. napus* genetic map of the AMDH population, derived from a cross between two French winter OSR varieties shows no significant difference in the number of markers mapped on the A and C genomes^42^. This would explain why the majority of QTL were on the A genome and the positive allelic effect on the A genome was predominantly from Ningyou7, whereas on the C genome Tapidor contributed the majority of the positive allelic effect (Tables S2, S3).

Using the new SNP map, there were 71 QTLs detected from the agar screen which compares to 38 detected using the previous map, and 22 as compared to 8 QTLs explained >10% of the phenotypic variation (Table S3). There was ~50% QTL integration across traits, treatments and systems, and integrating QTLs reduced the confidence interval by ~40%- after integration the mean interval was 3.7 cM, compared to 6 cM before (Tables S2, S3, S4). Therefore, a greater density SNP map and QTL integration has considerably improved QTL detection.

Under LP as compared to NP in the pouch system, root growth was greater in all traits, except primary root length (Data S1, S2). Similarly, in the agar system, lateral root number and density were greater and mean primary root length was shorter under LP compared to HP^27^, although in contrast to the pouch system lateral root length was also shorter at LP. Previous studies of *B. oleracea*^19^ and *B. napus*^20^ have also observed greater root length in plants with reduced P supply. A shorter primary root length and greater lateral root density has also been observed previously in response to P deficiency in *Arabidopisis*^43^,^44^. It could be argued that a reduced PRL in response to a low P supply is simply due to impaired growth, rather than being an adaptive response. For example, in *Arabidopsis* only half of the accessions tested showed a reduced primary root length in response to low Pi^45^. Furthermore, a shorter primary root length inevitably leads to a greater lateral root density because the latter is an inverse function of the former (e.g. LRD = LRN/PRL)^45^. Although, in *Arabidopsis* it was observed that an increase in lateral root density occurred along the branching zone of the primary root in response to low Pi availability, the branching zone is independent of the length of the primary root, thus lateral root density did increase independently of a reduced primary root length^44^. Therefore, although it is possible that a shorter primary root length is a general stress response, a greater lateral number and length suggests an adaptive/plastic response to nutrient deficiency.

As compared with Ningyou7, which is the P-efficient genotype, Tapidor had a longer primary root length but lower lateral root density at LP in both the pouch and wick system (Fig. 3; Data S2) and agar screen. The biomass of shoot and the shoot/root ratio of Ningyou7 were also significantly greater than Tapidor under LP under both culture systems (Data S2). Thus it appears that Ningyou7 trades a greater total root length for a greater lateral root density, and consequently is able to allocate more biomass to shoot rather than root, without any resource acquisition penalty. Likewise, in the field Ningyou7 has greater seed yield, seed weight and number of primary branches compared to Tapidor under low Pi^22^. The *Pup1* P efficiency locus is associated with a greater shoot/root ratio and it was suggested that maintaining a greater shoot biomass with less root is characteristic of P efficiency^46^. It has been suggested that for many crops and environments there is too much carbon invested in the roots^2^. Thus the QTLs associated with a shorter primary root length and greater lateral root density may promote greater shallow rooting and improve P use efficiency.

QTL integration found pleiotropic loci which controlled numerous root traits- including both lateral root length and number. In the agar system there were combined QTL on A3 for LRD + TLRL and on C9 for TRL + LRN, and in the pouch system on A3 for PRL + LRN (Tables S2, S3, S4). This pleiotropism is unsurprising given that these traits were positively correlated (Data S2). Although, interestingly, across the systems there was a combined QTL for LRD and MLRL on A9, this is surprising given that it was observed here and elsewhere that there is a negative relationship between MLRL and LRD^5^, suggesting that the same QTL is independently controlling both lateral root length and number.

It seems that there was no predominant effect of P treatment on the QTL detected, there were only two combined QTL from within a P treatment across the culture systems; PRL + LRN at on C5 and LRD and MLRL on A9, both at low P (Table S4). This is most likely explained by there being only a minimal P treatment effect compared to genotype effect, as indicated by the variance components analysis (Data S3). Although, under low P but not under high P, in both growth systems, the greatest number of QTL were for LRN, perhaps indicating the responsiveness of this trait to low P availability.

Most QTL for shoot and root biomass were on chromosomes A3 and A4, and most root traits also had QTL on these chromosomes (Figs 4 and 5). Likewise, most QTL for yield-related traits in the TNDH population in the field were on A3, and many of these co-localise with the QTL observed here^22^. Similarly, in a meta-analysis of QTL studies for yield related traits in *B. napus* it was found, post integration, that A3 had the most unique QTL^24^. Most QTL for root traits in *B. napus* grown in a paper culture system were on A3 and C3^20^. QTL for leaf P were identified in *B. rapa* on A3^47^. In *B. oleracea* it was observed that shoot P and P use efficiency (PUE) QTLs were mostly on C3 and C7^19^ and QTL for germination rate and percentage germination were found on C3^48^,^49^. It seems that chromosomes A3/C3 consistently influence vigour and yield-related traits, therefore the QTL observed for root length traits in the present study may be linked with general vigour.

Lateral root number and density had fewer QTL on A3 compared to other traits. When both culture systems are considered together, 78% of QTL for lateral root density and 84% of QTL for lateral root number were on chromosomes A4/C4, C2, C8, A9/C9 (Fig. 4). There was also an integrated QTL for LRD and LRN across the growth systems on chromosome A4 (Table S4). Furthermore, there are numerous examples of co-localised QTL on A4/C4 and C8/C9 for LRD and LRN, and it may be that homoeologous exchange has occurred in these chromosomes. For example, A4/C4 had co-localising QTL for LRN/LRD at LP in the pouch and HP in the agar systems, respectively. On C8/C9 there was co-localisation of QTL for LRN under HP and LP in agar, and for LRD/LRN under NP in the pouch and LP in the agar system, respectively (Tables S2, S3). Chromosome C8 has syntenic segments with A8 and C9, and C9 is largely collinear with A9 and C8^50^. Homoeologous exchange has previously been observed throughout the genome in *B. napus*^51^ but most extensively on chromosomes A1/C1, A3/C3, and A9/C9^52^,^53^,^54^. QTL related to leaf mineral concentration have also previously been identified on chromosomes A9/C9. In *B. oleracea*, a regulatory hotspot and a QTL were located on chromosome A9 for shoot P at low P, the regulatory hotspot on A9 had GO terms related to the cytoskeleton^55^- a structure intimately related to cell division and therefore perhaps lateral root initiation. In *B. oleracea* the most significant QTL for shoot concentrations of Ca, Mg, Zn and Na occurred on C9^56^. In a *B. napus* diversity panel, significantly associated SNP markers were observed on C9 and A9 for shoot concentrations of Ca, Mg, Zn and Na^57^, and in the same panel significantly associated GEMs (gene expression markers) for LRD as well as for shoot mineral concentrations were observed on A9 (Thomas *et al.*, 2016, unpublished).

In the present study QTL were found on chromosome A1 for root length under NP in the pouch system (Fig. 4). Likewise, a meta-analysis, pre-integration, observed that most QTL for yield-related traits in *B. napus* were on chromosome A1^24^. In *B. oleracea*, QTL for percentage germination and rate^48^ and hypocotyl upward growth^49^ were observed on chromosome C1. Also in *B. oleracea*, a QTL for physiological PUE (Phosphorus Use Efficiency) at optimal P and a regulatory hotspot responding to P availability, were located on chromosome A1, the hotspot had GO terms related to photosynthesis^55^. QTL for leaf P have also been identified on chromosome A1 in *B. rapa*^47^. Thus chromosome A1/C1, as well as A3, appear to be linked to vigorous growth under optimal P conditions.

Using the confidence interval found for the combined QTLs, a BLAST to the Darmor *B. napus* reference sequence^54^ identified 19 candidate genes related to root growth and plant genetic responses to low P availability, most of which were on chromosomes A3 and C9, including *LJRHL1-like* (*LRL2*) which controls root hair cell development, *AUXIN-INDUCED IN ROOT CULTURES 12* (*AIR12*) related to lateral root morphogenesis, and *Phosphate deficiency response 2* (*PDR2*) which mediates the developmental response of root meristems to phosphate availability. These candidates lay the foundation for a deeper dissection of the P starvation response mechanisms in *Brassica napus*.

## Methods

### Plant materials

Tapidor x Ningyou7 doubled haploid population (TNDH, N = 202)^58^. The DH population was derived from a cross between Tapidor and Ningyou7 by microspore culture. Ningyou7 was characterized as a P-efficient cultivar with better growth and higher P acquisition than Tapidor under low P (LP) and optimal P (OP or HP) conditions in pot culture and field trials^22^,^59^.

### SNP marker analysis and linkage map construction

Total genomic DNA of 202 genotypes of the TNDH population as well as the two parental lines was extracted from the young leaves by a modified CTAB (cetyl trimethylammonium bromide) method^60^. Five leaves from different individuals of each line were used to construct DNA bulks. DNA concentration in tubes was measured by electrophoresis through a conventional 2% agarose gel. 30 ng/μl, 50 ng/μl and 100 ng/μl λDNA (48,502 bp) was used as reference. The final DNA samples were diluted to 50 ng μL^−1^. The population and the parents were genotyped using the *Brassica* 60 k SNP BeadChip Array (Illumina Inc., USA). This array, which successfully assays 52,157 Infinium Type II SNP loci in *B. napus*, was developed using preferentially single-locus SNPs contributed from genomic and transcriptomic sequencing in genetically diverse *Brassica* germplasm^61^. DNA sample preparation, hybridization to the BeadChip, washing, primer extension and staining were performed according to the work flow described in the Infinium HD Assay Ultra manual. Imaging of the arrays was performed using an Illumina HiSCAN scanner after BeadChip washing and coating. Allele calling for each locus was performed using the GenomeStudio genotyping software (v2011, Illumina, Inc.). Positions of A-genome SNPs were provided by the array manufacturer, while C genome SNP source sequences were subjected to a BLAST search against the *B. oleracea* genome database (BRAD, http://brassicadb.org/) to locate chromosome positions (E value ≤ 1e-50).

The genotype data was visualized by the software Genomestudio (Illumina inc.). The genotype with missing data >5% were removed to reduce the mapping errors and avoid artificial exaggeration of map distances. From the genotypic data, marker pairs with zero recombination were assigned to the same bin. The representative SNP marker of a bin was selected to construct the genetic linkage map using the software packages of JoinMap 4.0^62^. The threshold for goodness-of-fit was set to ≤5.0 with a recombination frequency of <0.4 and a minimum logarithm of odds score of 1.0. Markers with a χ2 value of >3.0 were excluded in all genetic groups. Recombination frequencies were converted to centimorgans (cM) using the Kosambi method^63^ for map distance calculation according to the method described by Long *et al.*^64^. A reference genetic map was constructed with SNP bins being designated according to the best SNP marker in each bin. The last SNP-based map was integrated with the previous 798-map composed of SSR (single sequence repeat) and RFLP (restriction fragment length polymorphism) markers^22^,^27^,^64^. Then, Chi-square (χ^2^) tests were used for testing segregation distortion^65^. The markers with χ^2^ > 3.84 (*p* = 0.05) were designated segregation distorted markers (SD markers). The extreme SD markers which significantly deviated from the fit-line were abandoned^66^,^67^ (Fig. 1).

### High throughput root and shoot phenotyping

A ‘pouch and wick’ hydroponic-based HTP (high throughput phenotyping) system^31^ and image analysis procedure^5^ was deployed in this study. This system comprised growth pouches assembled from blue germination paper (SD7640; Anchor Paper Company, St Paul, MN, USA), re-cut to 24 × 30 cm and overlain with black polythene (Cransford Polythene Ltd, Woodbridge, UK). Along their shorter edges, the paper and polythene were clipped together using ‘bulldog’-type fold-back clips to each side of an acrylic bar (Acrylic Online, Hull, UK) giving 2 germination papers per pouch. The growth pouches were suspended above plastic drip trays, supported within lightweight aluminium/polycarbonate frames. Prior to sowing, the pouches were suspended above the nutrient solution for a minimum of 4 h to become fully saturated. A single seed was sown in the middle of the upper edge of each germination paper, by pressing the seed into the paper. Within each aluminium frame there were nine drip trays with 10 or 11 pouches per drip tray, arranged in three columns. A total of 4 frames were used in each experimental run, thus 96 pouches and 192 seedlings per frame, giving a potential sample size of 768 seedlings per run, within a single CE room. The CE room was 2.2 m wide, 3.3 m long, 3.0 m high, set to a 12 h photoperiod with 18/15 °C day/night temperatures and relative humidity of 60–80%. Photosynthetically Active Radiation (PAR; measured at plant height with a 190 SB quantum sensor; LI-COR Inc., Lincoln, NE, USA) was 207 μmol m^−2^ s^−1^, generated by 400 W white fluorescent lamps (HIT 400w/u/Euro/4K, Venture Lighting, Rickmansworth, UK). Drip trays were replenished with 500 mL of deionised water every 3 d. Fourteen days after sowing (DAS), the polythene sheets were removed from all pouches and images were taken of the germination paper and root system for downstream image analysis. Images were taken using a Digital Single Lens Reflex (DSLR) camera (Canon EOS 1100D, Canon Inc., Tokyo, Japan) with a focal length of 35 mm at a fixed height of 75 cm, using Canon software.

Seedlings of 199 of the genotypes and the parents (n = 4872) were screened at a ‘normal’ Pi concentration of 0.25 mM (NP treatment) using a ¼ strength Hoagland’s solution (No. 2 Basal Salt Mixture, Sigma Aldrich, Dorset, UK). Seedlings of all 202 genotypes and the parents (n = 5376) were also screened at a low Pi concentration of 0 mM (LP treatment) using a zero Pi Hoagland’s solution recipe, with K_2_SO_4_ added for balanced K. The pH of the nutrient solution was adjusted to be ~5.7 by HCl/NaOH, consistent with a pH of 5.8 in the NP treatment. Each genotype was grown in one experimental run, pouches were randomly allocated to a position within each column of each tank, giving ~24 replicates per run. Two additional oilseed rape lines were grown per run to serve as a reference for normalisation of the data, to account for run effects. Roots and shoots grown under the LP treatment were dried at 50 °C for 48 hours, and dry weights were taken (SDW, RDW) and total dry weight calculated (TDW, root + shoot). This data was not collected for the NP treatment due to unforeseen issues with sample storage.

### Root image analysis from the ‘pouch and wick’ system

The root images from the HTP system were renamed with each sample’s unique experimental design information using Bulk Rename Utility (Version 2.7.1.3, TGRMN Software, www.bulkrenameutility.co.uk). Images were cropped by reducing extraneous pixels on bulked images, using XnConvert (Version 1.66, www.xnconvert.com). Cropped images were analysed using RootReader2D^68^ (RR2D). First, a ‘batch process’ was carried out which automatically ‘thresholds’, ‘skeletonises’ and ‘builds segments’ of all images in bulk. The root system was then measured on individual images by placing a marker at the base and tip of the primary root. From these markers, RR2D automatically calculates primary root length (PRL), total lateral root length (TLRL) of all lateral roots and lateral root number (LRN). Total root length (TRL) = PRL + LRL, mean lateral root length (MLRL) = LRL/LRN and lateral root density (LRD) = LRN/PRL. A database was developed which integrated the experimental design information from the image name, with the RR2D measurements for each sample, using a programming script (2.7.10; Python Software Foundation, www.python.org).

The root traits of the TNDH population had been screened previously using an agar plate based system, at a Pi concentration of 0.625 mM (HP treatment) and 0 mM (LP treatment)^27^.

### Data analysis

The genotypic and non-genotypic variance components of root traits were calculated using a REML (REsidual Maximum Likelihood) procedure. In the REML model all factors were classed as random factors with no defined fixed factor^27^; [(Run/Frame/Column/Position/Paper side) + ([P]ext × Line)]. To acquire adjusted means the same model was used, however genotype was classed as a fixed factor and all other factors were random. Adjusted means were used for all statistical and QTL analyses. Pearson’s correlation coefficients calculated the relationship between traits within a P treatment and across P treatments. T-tests were performed to calculate differences in root growth between the P treatments and between the parents. All statistical analyses were conducted using GenStat (15^th^ Edition, VSN International Ltd, Hemel Hempstead, UK).

### QTL analyses and meta-analyses in the ‘pouch and wick’ and agar systems

QTL detection for root and shoot traits was carried out using the composite interval method (CIM), WinQTLCart (version 2.5)^69^. Walk speed was set as 1 cM. The estimated additive effect and the percentage of phenotypic variation explained by each putative QTL were obtained using the CIM model. A QTL was regarded as significant with manual input threshold value more than 11.5 (LOD threshold of 2.5). QTL support intervals were determined by 2-LOD intervals around the QTL peak.

QTLs for root and shoot traits across both treatments and culture systems were integrated in a meta-analysis using BioMercator^70^ (version 4.2). The Gerber & Goffinet meta-analysis model with the smallest AIC value was chosen for QTL integration. The principle of integration is that the peak position of component QTLs should be located within the confidence interval of integrated QTL.

## Additional Information

**How to cite this article**: Zhang, Y. *et al.* QTL meta-analysis of root traits in *Brassica napus* under contrasting phosphorus supply in two growth systems. *Sci. Rep.* **6**, 33113; doi: 10.1038/srep33113 (2016).

## Supplementary Material

Supplementary Table 1

Supplementary Table 2

Supplementary Table 3

Supplementary Table 4

Supplementary Data S1

Supplementary Data S2

Supplementary Data S3

## Figures and Tables

**Table 1:** Number of molecular markers, genetic distance, marker density in each linkage group of the *Brassica napus* TNDH 2041-map.

Linkage Group	Number of molecular Markers	Genetic Distance (cM)	Marker density (markers/cM)	SNP bin	SNP bin density (markers/cM)	Original markers
A1	142	89.4	1.63	132	1.48	10
A2	107	120.6	0.91	91	0.75	16
A3	158	131.8	1.44	121	0.92	37
A4	105	82.0	1.31	91	1.11	14
A5	105	112.7	0.83	94	0.83	11
A6	141	128.8	1.15	120	0.93	21
A7	131	104.9	1.30	108	1.03	23
A8	81	83.8	0.98	67	0.80	14
A9	153	139.7	1.10	102	0.73	51
A10	104	75.9	1.37	85	1.12	19
C1	97	92.6	1.06	90	0.97	7
C2	85	74.4	0.85	78	1.05	7
C3	127	142.4	0.84	104	0.73	23
C4	95	116.2	0.80	79	0.68	16
C5	62	133.4	0.47	52	0.39	10
C6	78	106.2	0.73	59	0.56	19
C7	83	93.5	0.76	67	0.72	16
C8	107	108.1	0.91	98	0.91	9
C9	80	141.4	0.57	60	0.42	20
A genome	1227	1069.6	1.18	1011	0.95	216
C genome	814	1008.3	0.76	687	0.68	127
Total(A + C)	2041	2077.9	0.97	1698	0.82	343

SNP bin, the smallest unit where chromosome recombination events happen. Original markers, SSR, RFLP and AFLP markers.
