# Supplementary material for: QTL meta-analysis of root traits in Brassica napus under contrasting phosphorus supply in two growth systems
Source: Sci Rep. 2016 Sep 14;6:33113. doi: 10.1038/srep33113 (PMC5021999; doi:10.1038/srep33113)
Supplement: Supplementary Table 1 [file srep33113-s1.doc]

**Manuscript title**： QTL meta-analysis of root traits in *Brassica napus* under contrasting phosphorus supply in two growth systems

**Author list**：Ying Zhang, Catherine L. Thomas, Jinxia Xiang, Yan Long, Xiaohua Wang, Jun Zou, Ziliang Luo, Guangda Ding, Hongmei Cai, Neil S. Graham, John P. Hammond, Graham King, Philip J. White, Fangsen Xu, Martin R. Broadley, Lei Shi, Jinling Meng

|  | 798 map | | | 1826 map | | | | 2109 map | | | | |
| --- | --- | --- | --- | --- | --- | --- | --- | --- | --- | --- | --- | --- |
| Linkage Group | No. of markers | Genetic Length (cM) | Marker density (markers/cM) | Bin | No. of markers | Genetic Length (cM) | Marker density (markers/cM) | No. of markers | SNP Bin | Original markers | Genetic Length (cM) | Marker density (markers/cM) |
| A1 | 48 | 98.3 | 0.49 | 139 | 754 | 76.5 | 1.82 | 146 | 136 | 10 | 89.5 | 1.63 |
| A2 | 39 | 96.0 | 0.41 | 97 | 507 | 111.7 | 0.87 | 110 | 92 | 18 | 120.6 | 0.91 |
| A3 | 81 | 130.5 | 0.62 | 156 | 1100 | 124.8 | 1.25 | 164 | 126 | 38 | 145.9 | 1.12 |
| A4 | 34 | 86.9 | 0.39 | 113 | 701 | 70.5 | 1.60 | 110 | 92 | 18 | 84.2 | 1.31 |
| A5 | 45 | 115.4 | 0.39 | 96 | 616 | 96.0 | 1.00 | 106 | 94 | 12 | 127.4 | 0.83 |
| A6 | 55 | 134.5 | 0.41 | 126 | 599 | 110.1 | 1.14 | 148 | 125 | 23 | 128.9 | 1.15 |
| A7 | 52 | 113.0 | 0.46 | 113 | 919 | 73.8 | 1.53 | 137 | 111 | 26 | 105.5 | 1.30 |
| A8 | 31 | 92.3 | 0.34 | 68 | 283 | 65.3 | 1.04 | 82 | 67 | 15 | 84.0 | 0.98 |
| A9 | 81 | 143.1 | 0.57 | 102 | 605 | 120.9 | 0.84 | 154 | 101 | 53 | 139.8 | 1.10 |
| A10 | 35 | 88.5 | 0.40 | 85 | 359 | 64.2 | 1.32 | 104 | 85 | 19 | 75.9 | 1.37 |
| C1 | 36 | 72.9 | 0.49 | 94 | 828 | 91.7 | 1.03 | 99 | 91 | 8 | 93.8 | 1.06 |
| C2 | 31 | 80.7 | 0.38 | 80 | 604 | 87.8 | 0.91 | 90 | 79 | 11 | 105.7 | 0.85 |
| C3 | 57 | 180.0 | 0.32 | 110 | 998 | 150.3 | 0.73 | 139 | 110 | 29 | 164.8 | 0.84 |
| C4 | 40 | 141.6 | 0.28 | 103 | 2298 | 113.3 | 0.91 | 104 | 86 | 18 | 129.3 | 0.80 |
| C5 | 20 | 88.4 | 0.23 | 52 | 377 | 125.6 | 0.41 | 63 | 52 | 11 | 133.7 | 0.47 |
| C6 | 38 | 99.0 | 0.38 | 60 | 569 | 90.7 | 0.66 | 78 | 59 | 19 | 106.2 | 0.73 |
| C7 | 26 | 104.0 | 0.25 | 70 | 469 | 109.2 | 0.64 | 87 | 70 | 17 | 114.9 | 0.76 |
| C8 | 23 | 81.4 | 0.28 | 102 | 721 | 116.5 | 0.88 | 108 | 98 | 10 | 118.8 | 0.91 |
| C9 | 26 | 106.6 | 0.24 | 60 | 305 | 119.0 | 0.50 | 80 | 60 | 20 | 141.4 | 0.57 |
| A genome | 501 | 1098.4 | 0.46 | 1095 | 6443 | 913.7 | 1.20 | 1261 | 1029 | 232 | 1101.6 | 1.14 |
| C genome | 297 | 954.6 | 0.31 | 731 | 7169 | 1004.2 | 0.73 | 848 | 705 | 143 | 1108.6 | 0.76 |
| Total (A+C) | 798 | 2053.1 | 0.39 | 1826 | 13612 | 1917.9 | 0.95 | 2109 | 1734 | 375 | 2210.2 | 0.95 |

**Supplementary table 1. Number of molecular markers, genetic distance and marker density on each linkage group of the *Brassica napus* TNDH 798-map, 1826-map and 2109-maps.** Original markers means the traditional molecular markers; SSR, RFLP, AFLP.
