# Supplementary material for: QTL meta-analysis of root traits in Brassica napus under contrasting phosphorus supply in two growth systems
Source: Sci Rep. 2016 Sep 14;6:33113. doi: 10.1038/srep33113 (PMC5021999; doi:10.1038/srep33113)
Supplement: Supplementary Table 2 [file srep33113-s2.doc]

**Manuscript title**： QTL meta-analysis of root traits in *Brassica napus* under contrasting phosphorus supply in two growth systems

**Author list**：Ying Zhang, Catherine L. Thomas, Jinxia Xiang, Yan Long, Xiaohua Wang, Jun Zou, Ziliang Luo, Guangda Ding, Hongmei Cai, Neil S. Graham, John P. Hammond, Graham King, Philip J. White, Fangsen Xu, Martin R. Broadley, Lei Shi, Jinling Meng

| **P condition** | **Chr.** | **Component QTL** | **Combined QTL** | **LOD** | **R^2^ (%)** | **Peak position (cM)** | **CI (cM)** | **Add. Effect** |
| --- | --- | --- | --- | --- | --- | --- | --- | --- |
| **NP** | A1 |  | ***uq.P_NP_A1_1*** | **4.0** | **8.1** | **45.5** | **43.6 - 47.3** |  |
|  |  | ***qTLRL_NP_A1a*** |  |  | **7.6** | **45.2** | **41.7 - 46.9** | **+** |
|  |  | ***qTRL_NP_A1a*** |  |  | **8.7** | **45.7** | **41.7 - 46.9** | **+** |
|  |  |  | ***uq.P_NP_A1_2*** | **5.0** | **10.4** | **52.1** | **50.0 - 54.2** |  |
|  |  | ***qTRL_NP_A1b*** |  |  | **10.2** | **52.1** | **46.9 - 53.3** | **+** |
|  |  | ***qTLRL_NP_A1b*** |  |  | **10.6** | **52.1** | **47.6 - 53.3** | **+** |
|  | A3 |  | ***uq.P_NP_A3_1*** | **4.5** | **10.1** | **15.0** | **8.1 - 21.8** |  |
|  |  | ***qPRL_NP_A3a*** |  |  | **12.4** | **13.1** | **1.1 - 17.8** | **-** |
|  |  | ***qLRN_NP_A3a*** |  |  | **7.9** | **18.8** | **2.5 - 26.3** | **-** |
|  |  | *qPRL_NP_A3b* |  | 3.4 | 6.8 | 28.8 | 27.8 - 29.9 | - |
|  | A10 | *qLRD_NP_A10a* |  | 3.6 | 7.3 | 37.1 | 35.4 - 37.7 | - |
|  |  | *qTRL_NP_A10a* |  | 2.5 | 5.6 | 50.6 | 45.4 - 51.4 | - |
|  |  | *qTLRL_NP_A10a* |  | 3.5 | 6.9 | 53.0 | 52.2 - 55.6 | - |
|  | C4 | *qLRN_NP_C4a* |  | 2.7 | 5.3 | 81.3 | 80.4 - 83.4 | + |
|  |  | *qLRN_NP_C4b* |  | 4.5 | 8.8 | 91.4 | 91.0 - 95.7 | + |
|  |  | *qLRN_NP_C4c* |  | 3.1 | 6.0 | 106.7 | 102.5 - 111.5 | - |
|  | C9 | *qLRD_NP_C9a* |  | 3.6 | 7.1 | 61.4 | 60.6 - 61.9 | + |
| **LP** | A3 | *qPRL_LP_A3a* |  | 3.2 | 6.3 | 33.7 | 28.8 - 43.5 | - |
|  | A4 | *qLRN_LP_A4a* |  | 2.9 | 4.8 | 31.7 | 31.0 - 35.8 | - |
|  |  | *qLRN_LP_A4b* |  | 4.2 | 6.8 | 39.2 | 35.8 - 42.4 | - |
|  |  | *qLRN_LP_A4c* |  | 3.3 | 5.5 | 45.0 | 44.1 - 48.1 | - |
|  |  | *qLRD_LP_A4a* |  | 3.2 | 5.2 | 59.0 | 58.1 - 60.0 | - |
|  |  |  | ***uq.P_LP_A4_5*** | **3.2** | **6.0** | **61.7** | **60.9 - 62.4** |  |
|  |  | ***qTDW_LP_A4a*** |  |  | **5.8** | **61.2** | **60.7-64.0** | **-** |
|  |  | ***qSDW_LP_A4a*** |  |  | **6.2** | **61.8** | **60.9-62.6** | **-** |
|  |  |  | ***uq.P_LP_A4_6*** | **4.0** | **7.3** | **71.0** | **66.2 - 75.8** |  |
|  |  | ***qTDW_LP_A4b*** |  |  | **6.4** | **71.0** | **64.0-80.0** | **-** |
|  |  | ***qSDW_LP_A4b*** |  |  | **8.2** | **71.0** | **67.9-80.0** | **-** |
|  | A5 | *qMLRL_LP_A5a* |  | 4.2 | 8.2 | 0.0 | 0.0 - 8.0 | - |
|  |  |  | ***uq.P_LP_A5_2*** | **4.9** | **9.2** | **40.0** | **38.4 - 41.6** |  |
|  |  | ***qPRL_LP_A5a*** |  |  | **8.8** | **40.0** | **37.8-42.5** | **-** |
|  |  | ***qSDW_LP_A5a*** |  |  | **9.7** | **40.0** | **37.8-42.2** | **-** |
|  |  | *qPRL_LP_A5b* |  | 3.7 | 7.3 | 49.5 | 48.1 - 50.3 | - |
|  |  | *qTDW_LP_A5a* |  | 4.7 | 8.9 | 53.8 | 53.5 - 54.5 | - |
|  |  | *qRDW_LP_A5a* |  | 3.8 | 7.3 | 56.4 | 54.5 - 63.0 | - |
|  |  | *qTDW_LP_A5b* |  | 3.3 | 6.3 | 65.2 | 63.8 - 66.9 | - |
|  | A6 | *qRDW_LP_A6a* |  | 3.5 | 6.9 | 18.1 | 16.8 - 18.2 | - |
|  |  | *qTRL_LP_A6a* |  | 2.7 | 5.2 | 21.5 | 20.9 - 25.4 | - |
|  | A7 |  | ***uq.P_LP_A7_1*** | **4.9** | **9.1** | **67.3** | **65.8 - 68.8** |  |
|  |  | ***qSDW_LP_A7a*** |  |  | **10.8** | **67.1** | **65.7-73.2** | **-** |
|  |  | ***qTDW_LP_A7a*** |  |  | **7.5** | **67.3** | **65.7-69.0** | **-** |
|  |  | *qTDW_LP_A7b* |  | 3.4 | 6.4 | 77.2 | 76.3 - 77.3 | - |
|  |  | *qRDW_LP_A7a* |  | 2.6 | 5.9 | 101.3 | 90.6 - 103.3 | - |
|  | A9 |  | ***uq.P_LP_A9_1*** | **2.8** | **6.0** | **30.4** | **26.5 - 34.2** |  |
|  |  | ***qTLRL_LP_A9a*** |  |  | **6.0** | **30.1** | **26.1 – 35.2** | **+** |
|  |  | ***qTRL_LP_A9a*** |  |  | **5.9** | **31.1** | **26.1-40.6** | **+** |
|  |  | *qMLRL_LP_A9a* |  | 5.8 | 11.7 | 104.8 | 104.3 - 107.5 | - |
|  |  | *qMLRL_LP_A9b* |  | 6.3 | 12.5 | 112.8 | 111.3 - 113.6 | - |
|  |  | *qLRN_LP_A9a* |  | 4.0 | 6.8 | 115.3 | 115.0 - 117.0 | + |
|  |  | *qLRN_LP_A9b* |  | 6.3 | 10.6 | 123.9 | 122.9 - 124.5 | + |
|  | C2 |  | ***uq.P_LP_C2_1*** | **3.7** | **6.6** | **5.9** | **4.1 - 7.6** |  |
|  |  | ***qLRD_LP_C2a*** |  |  | **4.7** | **5.3** | **3.1 - 7.5** | **+** |
|  |  | ***qLRN_LP_C2a*** |  |  | **8.6** | **6.9** | **3.5 - 9.4** | **+** |
|  |  |  | ***uq.P_LP_C2_2*** | **5.0** | **8.3** | **15.9** | **13.9 - 17.9** |  |
|  |  | ***qLRD_LP_C2b*** |  |  | **6.4** | **15.9** | **15.3 - 21.6** | **+** |
|  |  | ***qLRN_LP_C2b*** |  |  | **10.2** | **15.9** | **15.3 - 20.6** | **+** |
|  |  | *qMLRL_LP_C2a* |  | 5.8 | 12.4 | 19.2 | 16.2 - 22.9 | - |
|  |  |  | ***uq.P_LP_C2_4*** | **4.0** | **6.8** | **24.7** | **23.9 - 25.5** |  |
|  |  | ***qLRD_LP_C2c*** |  |  | **4.7** | **24.7** | **22.5 - 25.7** | **+** |
|  |  | ***qLRN_LP_C2c*** |  |  | **8.6** | **24.7** | **23.7 - 25.6** | **+** |
|  |  | *qMLRL_LP_C2b* |  | 3.7 | 7.1 | 34.7 | 34.3 - 35.7 | + |
|  | C4 |  | ***uq.P_LP_C4_1*** | **2.7** | **4.6** | **42.5** | **39.1 - 45.9** |  |
|  |  | ***qRDW_LP_C4a*** |  |  | **5.1** | **42.5** | **38.3-53.8** | **+** |
|  |  | ***qLRN_LP_C4a*** |  |  | **4.1** | **42.5** | **40.3 - 47.8** | **+** |
|  |  | *qSDW_LP_C4a* |  | 3.1 | 5.5 | 63.4 | 60.6 - 70.3 | + |
|  | C5 | *qMLRL_LP_C5a* |  | 3.3 | 6.3 | 16.2 | 10.9 - 17.8 | + |
|  |  | *qLRN_LP_C5a* |  | 3.4 | 6.0 | 48.7 | 44.2 - 53.0 | - |
|  | C9 |  | ***uq.P_LP_C9_1*** | **3.6** | **7.3** | **9.4** | **4.6 - 14.2** |  |
|  |  | ***qTLRL_LP_C9a*** |  |  | **7.3** | **9.4** | **0.0 - 17.6** | **+** |
|  |  | ***qTRL_LP_C9a*** |  |  | **7.3** | **9.4** | **6.0-17.6** | **+** |
|  |  | *qLRD_LP_C9a* |  | 6.7 | 12.8 | 18.6 | 14.7 - 22.1 | + |
|  |  |  | ***uq.P_LP_C9_3*** | **6.5** | **11.8** | **27.6** | **22.5 - 32.7** |  |
|  |  | ***qLRN_LP_C9a*** |  |  | **6.0** | **26.1** | **22.1 - 40.5** | **+** |
|  |  | ***qLRD_LP_C9b*** |  |  | **17.5** | **28.3** | **24.0 - 36.2** | **+** |

**Supplementary Table 2. Component and integrated QTL (in bold) for root and shoot traits in the TNDH population grown for 14 days at low Pi (LP, 0 mM Pi) and normal Pi (NP, 0.25 mM Pi) in a ‘pouch and wick’ system.** The QTL analysis was based on the new high-density 2041 map constructed in this study. TRL = total root length, PRL = primary root length, TLRL = total lateral root length, LRN = lateral root number, LRD = lateral root density, SDW = shoot dry weight, RDW = root dry weight, TDW = total dry weight, Chr. = chromosome, R^2^ = the explained phenotypic variation, CI = confidence interval at P=0.05, Add. Effect = additive effect; + allele from the parent Tapidor, - allele from the parent Ningyou7.
